# Workplace Secondhand Smoke Exposure in the U.S. Trucking Industry

**DOI:** 10.1289/ehp.0900892

**Published:** 2009-10-05

**Authors:** Yueh-Hsiu Chiu, Jaime E. Hart, Donna Spiegelman, Eric Garshick, Thomas J. Smith, Douglas W. Dockery, S. Katharine Hammond, Francine Laden

**Affiliations:** 1 Exposure, Epidemiology, and Risk Program, Department of Environmental Health, Harvard School of Public Health, Boston, Massachusetts, USA; 2 Channing Laboratory, Brigham and Women’s Hospital and Harvard Medical School, Boston, Massachusetts, USA; 3 Department of Epidemiology and; 4 Department of Biostatistics, Harvard School of Public Health, Boston, Massachusetts, USA; 5 Pulmonary and Critical Care Medicine Section, Veterans Affairs Boston Healthcare System, Boston, Massachusetts, USA; 6 School of Public Health, University of California–Berkeley, Berkeley, California, USA

**Keywords:** personal sampling, secondhand smoke, self-reported exposure, smoking policy, trucking industry, vapor-phase nicotine, workplace exposure

## Abstract

**Background:**

Although the smoking rate in the United States is declining because of an increase of smoke-free laws, among blue-collar workers it remains higher than that among many other occupational groups.

**Objectives:**

We evaluated the factors influencing workplace secondhand smoke (SHS) exposures in the U.S. unionized trucking industry.

**Methods:**

From 2003 through 2005, we measured workplace SHS exposure among 203 nonsmoking and 61 smoking workers in 25 trucking terminals. Workers in several job groups wore personal vapor-phase nicotine samplers on their lapels for two consecutive work shifts and completed a workplace SHS exposure questionnaire at the end of the personal sampling.

**Results:**

Median nicotine level was 0.87 μg/m^3^ for nonsmokers and 5.96 μg/m^3^ for smokers. As expected, smokers experienced higher SHS exposure duration and intensity than did nonsmokers. For nonsmokers, multiple regression analyses indicated that self-reported exposure duration combined with intensity, lack of a smoking policy as reported by workers, having a nondriver job, and lower educational level were independently associated with elevated personal nicotine levels (model *R*^2^ = 0.52). Nondriver job and amount of active smoking were associated with elevated personal nicotine level in smokers, but self-reported exposure, lack of a smoking policy, and lower educational level were not.

**Conclusions:**

Despite movements toward smoke-free laws, this population of blue-collar workers was still exposed to workplace SHS as recently as 2005. The perceived (reported by the workers), rather than the official (reported by the terminal managers), smoking policy was associated with measured SHS exposure levels among the nonsmokers. Job duties and educational level might also be important predictors of workplace SHS exposure.

There is a well-described association between adult exposures to secondhand smoke (SHS) and several adverse health effects, including chronic respiratory symptoms, lung cancer, and cardiovascular disease [Barnoya and Glantz 2005; Blanc et al. 1999; Centers for Disease Control and Prevention (CDC) 2006; Ho et al. 2007; Jindal et al. 1994; National Cancer Institute 1999; Panagiotakos et al. 2002; Raupach et al. 2006]. Recently, smoking rates have been declining among U.S. adults (CDC 2005, 2006; Pirkle et al. 2006; Wingo et al. 1999), particularly because of an increase in the number of states and cities that have implemented smoking bans, and this change may have affected locations and levels of SHS exposure in the workplace, including a shift from indoor to outdoor areas as workplace smoking policies change (Eisner et al. 2001).

Although declining, the smoking prevalence of blue-collar workers remains higher than that of many other occupational groups (Bang and Kim 2001; Nelson et al. 1994), suggesting that this is a population with high potential for SHS exposure at work (Wortley et al. 2002). In the National Health Interview Survey that included > 290,000 U.S. civilians, the major blue-collar worker groups (e.g., construction labors, motor vehicle operators, freight handlers, assemblers, mechanics) had a large decrease in the annual smoking prevalence in the 1997–2004 survey period relative to the 1987–1994 survey period, but still had a higher smoking rate (> 30%) compared with all workers combined (24.5%) in the 1997–2004 period (Lee et al. 2007). In addition, blue-collar workers are less likely to report a smoke-free worksite than are white-collar workers (Gerlach et al. 1997; Plescia et al. 2005).

We evaluated SHS exposures in the U.S. unionized trucking industry. Our exposure assessment included a self-administered questionnaire and monitoring of passive vapor-phase nicotine exposure among workers throughout the United States. The objectives of this study were to identify the factors influencing workplace exposure to SHS and to validate self-reported SHS exposure using personal vapor-phase nicotine levels.

## Materials and Methods

### Study subjects

We visited 25 trucking terminals (work locations) throughout the United States between 2003 and 2005. The terminals were randomly selected to represent the terminals with at least 100 employees from three large unionized trucking companies. We invited workers to participate in personal sampling and to complete a health questionnaire, which included questions on SHS exposure. The detailed job categories in the industry and their usual job duties are described elsewhere (Smith et al. 2006). In brief, the job titles are categorized mainly as long-haul driver (driving between cities), pickup/delivery (P&D) driver (local driving within cities), dock worker (moving freight within the terminal), combination worker (workers who both work on the dock and drive P&D trucks), mechanic (repairing tractors and trailers), hostler (moving trucks in the terminal yard), and clerk (office worker). This study was part of an overall assessment of occupational particulate exposures in the trucking industry (Smith et al. 2006). The main study began in 2002, and assessment of SHS exposure for these volunteers began 1 year later. These participants were asked to wear a passive personal nicotine sampler for two consecutive work shifts on 2 consecutive days and to complete a questionnaire at the end of personal monitoring. The protocol was approved by the human subjects committees at the Brigham and Women’s Hospital, the Harvard School of Public Health, and the Veterans Affairs Boston Healthcare System, and each participant provided informed consent before participating.

### Personal nicotine monitoring

Because of the predominance of nicotine in the vapor phase of SHS (> 90%) and because cigarette smoke is the only likely source of nicotine in the work environment (Hammond and Leaderer 1987), we used a passive monitor to obtain a quantitative measurement of vapor-phase nicotine in the breathing zone. The detailed sampler design, sampling methods, and validation have been described previously (Hammond and Leaderer 1987; Hammond et al. 1987). The monitor is constructed from a modified 37-mm-diameter polystyrene air sampling cassette, with a clip attached to the bottom so that the badge can be easily attached on the worker’s clothes. The filter within was treated by saturating it with an aqueous solution of 4% sodium bisulfate and 5% ethanol and allowing it to dry before placing it in the cassette. During the work shift the cover is removed from the cassette to expose the treated filter. At the end of each work shift, the badges were checked for damage and the cover replaced and sealed to prevent further exposure to SHS. None of the sampling badges were damaged.

The badges were analyzed by desorption of the nicotine and quantification by gas chromatography with nitrogen-selective detection. The limit of detection (LOD) of the passive monitor was < 0.01 μg, and the coefficient of variability for replicate analysis was 0.11 (Hammond and Leaderer 1987). In our study, 95.5% of the samples were above the LOD. We then calculated personal nicotine concentration by dividing the amount of nicotine by the volume of air estimated to passively diffuse through the sampler [monitoring duration multiplied by the effective sampling rate of the nicotine badge of 2.4 × 10^−5^ m^3^/min at 23°C (Hammond and Leaderer 1987)].

### SHS exposure questionnaire and smoking policy

For each participant, we collected self-reported information on demographic characteristics (age, sex, race, educational level, residence), work history (job title, work shift, employment history), history of smoking, and SHS exposure (at work, at home, in social situations, exposure history). The questionnaire focused on exposure in the 2 days before questionnaire completion (consistent with the personal sampling days), including the duration (time spent in areas at work where smoking is allowed) and the intensity (number of people smoking in those areas) of SHS exposure. We also asked the workers about exposure-related symptoms, as well as the smoking policy at the workplace (workers’ “perceived” smoking policy) and policy enforcement. If smoking was allowed in a specific area, the questionnaire further ascertained the location and size of the restricted area. In addition, we also collected information on the smoking policy from the terminal managers (considered as the “official” smoking policy). The smoking policy was categorized as “no policy/no restrictions (smoking allowed anywhere),” “indoor restricted area policy (smoking allowed in certain designated indoor areas),” and “outdoor only policy (smoking allowed only at outdoor areas).” Information regarding state- or county-level workplace smoking policy and the effective dates were obtained from the U.S. Tobacco Control Laws Database by the American Nonsmokers’ Rights Foundation (2009). We first identified the governmental workplace smoking policy at the county level, and for those terminals located in counties without a workplace smoking policy, we used the data at the state level.

### Statistical analysis

We calculated the arithmetic mean, SD, geometric mean (GM), geometric SD (GSD), median, 25th/75th percentiles, and range for the personal nicotine concentration, minutes spent in a smoking area, and numbers of smokers in the smoking area. Because drivers spend a large amount of time away from the terminals, job title was categorized into “driver” (P&D and long-haul drivers) and “nondriver” (all other job titles). We used the Wilcoxon rank-sum test for non-normally distributed continuous variables to assess the significance of differences observed between nonsmokers and smokers. The raw nicotine concentrations for each category of the questionnaire variables were stratified by smoking status. Then the regression residual method (Willett and Stampfer 1986) was used to adjust for clustering of levels within trucking terminals; these terminal-adjusted residuals were then compared by the Wilcoxon rank-sum test or the Kruskal–Wallis test, as appropriate.

To identify the factors influencing personal nicotine concentration, we first used univariate regression analyses to obtain the crude parameter estimator of nicotine concentration for each potential predictor. Because the residuals of these multiple regression models were right skewed, we used the robust variance for statistical inference (Huber 1967; White 1980). We considered variables that were either logically meaningful, potential confounders or statistically significant in univariate analyses. We accounted for the correlations of observations within a trucking terminal and during a sampling trip by including indicator variables for each terminal. Indicator variables were also used to account for missing information on covariates. We defined influential observations as those with a critical value of the Cook’s *D* statistic greater than 4/*n*, where *n* is the number of observations (Cook and Weisberg 1982). Finally, we conducted sensitivity analyses to compare the results of the models with and without the influential observations, and to compare the models including all the participants and including only those participants with complete information on all predictors. We also performed the regression analyses on the log-transformed scale of nicotine levels. In addition, an analysis including both fixed and random effects of terminal was conducted. All analyses were performed separately for smokers and nonsmokers, using the SAS statistical package (version 9.1.3; SAS Institute Inc., Cary, NC).

## Results

### Comparisons between smokers and nonsmokers

A total of 264 workers participated in the study, 203 (76.9%) nonsmokers and 61 (23.1%) smokers. Table 1 presents participant characteristics by smoking status. In our study population, the demographic and health-related characteristics were similar for smokers and nonsmokers, except that nonsmokers were more likely to report being bothered by tobacco smoke and to report irritation of the eye, nose, or throat after exposure to cigarette smoke in the previous 7 days.

The median (25th to 75th percentile) nicotine level was 0.87 (0.38–2.26) μg/m^3^ for nonsmokers and 5.96 (2.49–14.31) μg/m^3^ for smokers. The mean ± SD and GM (GSD) nicotine concentration for nonsmokers were 2.35 ± 5.41 μg/m^3^ and 0.97 μg/m^3^ (3.63), respectively, and for smokers were 13.63 ± 20.26 μg/m^3^ and 6.34 μg/m^3^ (3.82), respectively. As expected, concentrations were significantly higher for smokers than for nonsmokers (*p* < 0.0001 for all workers), regardless of the perceived smoking policies (*p* < 0.0001 for both “indoor restricted area policy” and “outdoor only policy” groups) and driver/nondriver status (*p* < 0.0001 for both nondrivers and drivers). The nicotine level difference between smokers and nonsmokers was not statistically significant for workers reporting “no policy” (*p* = 0.15), probably because of small numbers of people in this category. Self-reported SHS, including exposure duration (*p* = 0.02) and intensity (*p* = 0.01), was also statistically significantly different between nonsmokers and smokers. However, when we stratified the data by driver/nondriver status, both the self-reported exposure duration and intensity were significantly greater for smokers than for nonsmokers only among nondrivers (*p* = 0.02 and *p* = 0.03, respectively), whereas these variables were not statistically different among the drivers (*p* = 0.83 and *p* = 0.30, respectively).

### Personal nicotine levels by participant characteristics

Table 2 summarizes nicotine concentrations among nonsmokers and current smokers. As expected, for both nonsmokers and smokers, nicotine concentrations were higher for workers who spent more time in smoking areas with more people smoking in those areas in the previous 2 days (corresponding to the sampling period). Additionally, nicotine levels were higher for workers working at the terminals without a smoking policy, determined either by workers’ self-reports or the official smoking policies, as well as the state or county law, than for those who worked at the terminals with some smoking restrictions in place. Although only three nonsmokers and two smokers reported “no policy/no restrictions,” these workers had much higher nicotine concentrations than did other participants. Nonparametric tests after adjusting for trucking terminal also suggested that job title and work shift are associated with measured nicotine levels in nonsmokers.

Table 3 presents the crude and adjusted regression models of personal nicotine levels among the nonsmokers. We removed the five influential people identified by Cook’s *D* in all of these analyses (nicotine concentrations: 0.60, 15.32, 24.49, 36.96, and 56.89 μg/m^3^). These workers were from four different trucking terminals, and two of them were drivers; we found no obvious patterns in perceived smoking policy, work shift, or age among these workers. The results from the multivariable-adjusted model (*R*^2^ = 0.52) indicated that spending at least 30 min in an area where smoking was allowed with three or more smokers present, “no policy” as reported by the worker, nondriver status, night shift, and lower educational level were statistically significantly associated with elevated nicotine levels. However, the presence of an official smoking policy or a state/county workplace smoking ban was not a significant predictor. In sensitivity analyses restricted to people with complete information on all important potential predictors (*n* = 136), the results did not materially change. Similarly, the conclusions from the model including the influential points (*n* = 203) were not different with the exception that job title and work shift became nonsignificant and the *R*^2^ decreased to 0.20. Results were materially unchanged when we log-transformed the nicotine levels or when we included both fixed and random effects of terminal in the models (data not shown).

For smokers, nicotine concentrations were statistically significantly predicted by job title (7.97 μg/m^3^, *p* = 0.05, nondriver vs. driver), work shift (40.6 μg/m^3^, *p* < 0.0001, evening shift vs. others), and the number of cigarettes smoked in the previous 2 days (17.9 μg/m^3^, *p* < 0.0001, > 20 cigarettes vs. ≤ 20 cigarettes), after adjusting for trucking terminal and age (*R*^2^ = 0.72). However, self-reported exposure duration and intensity, lower educational level, and lack of a perceived, official, or state/county smoking policy were not associated with elevated measured personal nicotine levels.

### Relationships between self-reported and official smoking policies

Twenty-one of the 25 terminals were located in states or counties where a workplace smoking ban had not been enacted at the time of our sampling visit. However, 19 of these terminals already had an “official” smoking restriction policy (5 with “indoor restricted area policy” and 14 with “outdoor only policy”), as reported by their managers. The four terminals located in the states or counties where workplace smoking bans were in effect had an official smoking policy (one with “indoor restricted area policy” and three with “outdoor only policy”).

Although most of the terminals (23 of 25) had an “official” policy, the smoking policy reported by the workers (“perceived”) and the smoking policy provided by the managers (“official”) agreed only half the time (49% for nonsmokers, 52% for smokers). Eighteen nonsmokers and five smokers reported that they did not know the smoking policy at their workplace. Among those who reported that they knew the smoking policy, five workers (from four terminals: three nonsmokers and two smokers) reported “no policy,” whereas 20 workers worked at terminals without an “official” policy. Among those five workers reporting “no policy,” only one was actually working at a terminal without any official smoking policy. Two of the three nonsmokers who reported “no policy” and 63% of those who reported “indoor restricted area policy” were working at terminals with an official “outdoor only policy,” and 24% of the nonsmokers who perceived “outdoor only policy” were in fact working at terminals where the official policy was “indoor restricted area policy.” Both of the smokers who perceived “no policy” were working at the terminals where the official policy was “indoor restricted area policy,” and 55% of the smokers who thought that their workplace had an “indoor restricted area policy” were actually working at terminals where the official policy was “outdoor only.” Among the nonsmokers, 23% reported that the smoking policy they reported has “always/often” been enforced at the workplace, 27% “sometimes/rarely,” and 17% “never”; 34% stated that they did not know about the enforcement. Among the smokers, 10% reported “always/often” smoking enforcement, 21% “sometimes/rarely,” 16% “never,” and 52% “don’t know.”

## Discussion

We used a self-administered questionnaire to explore predictors of workplace SHS exposure, as measured by personal exposure to vapor-phase nicotine, for a population of trucking industry workers. For nonsmokers, the self-reported duration of time spent in smoking-allowed areas, combined with the average number of people smoking in those areas, was a positive predictor of measured nicotine concentrations, suggesting that the self-reports of SHS exposure among the nonsmokers in this population is relatively reliable. In addition, the absence of a workplace smoking policy (as reported by the workers) was significantly associated with elevated personal nicotine levels among the nonsmokers. However, these factors were not statistically significant predictors of personal nicotine levels among the smokers. Not surprisingly, both smoking and nonsmoking drivers had statistically significantly lower (but detectable) levels of nicotine compared with nondrivers, after controlling for active smoking, because drivers in general spend less time with other workers than nondrivers do. Note that although drivers spend most of their work day alone in their truck cabs, nonsmoking drivers still have an opportunity to be exposed to SHS at the trucking terminals, at delivery docks, and on breaks. The “official” smoking policy, as reported by the terminal managers, and the existence of a state or county smoking ban were not associated with nicotine levels for either nonsmokers or smokers. The “perceived” smoking policy reported by the workers and the official policy agreed only about half of the time, implying either that enforcement and compliance of the smoking policy in these workplaces were poor or that in fact the official policy was not well correlated with actual exposure. It is interesting to note that, among the workers who worked at the terminals without any official smoking policy (*n* = 20), most of them (95%) perceived that their workplace has some restrictions on cigarette smoking.

Although smokers are certainly exposed to SHS from their own cigarettes and the other smokers around them, it is difficult to tease out predictors of these exposures because of the strong effects of active smoking. In a study of SHS in the home, Leaderer and Hammond (1991) found a linear relationship between self-reported number of cigarettes smoked and area levels of vapor-phase nicotine. In our data, the amount of self-reported active smoking explained a large proportion of the variation in nicotine concentrations (*R*^2^ = 0.44 in the model including only the number of cigarettes smoked in the previous 2 days, age, and trucking terminal) and may have masked the smaller effects of workplace smoking policy or time spent with other smokers.

We did not find a statistically significant difference in nicotine concentration between the nonsmokers who reported an “indoor restricted area policy” and those who reported an “outdoor only policy” (Table 3), possibly because of the low power due to small sample size. In addition, this might be due to errors in the reporting of policy or the possibility of differences in behavior of smokers dependent on the policy that would affect the actual exposure levels. Another explanation is that indoor air might be contaminated by smoking right at doorways.

Blue-collar workers and service workers are more likely to be exposed to workplace SHS than are other worker groups (CDC 2006; U.S. Environmental Protection Agency 1992), likely due to higher smoking prevalence in these groups and the lower prevalence of smoke-free workplace policies. In the nationwide Current Population Survey (CPS) conducted in 1992–1993, > 70% of blue-collar workers and > 60% of service workers worked in workplaces that had no smoking restriction policy, a significantly higher percentage than among workers in other industries (Gerlach et al. 1997). In a study examining trends in smoke-free workplace policies from 1992 through 2002 in North Carolina, Plescia et al. (2005) found that blue-collar and service workers, especially males, were less likely to work in a smoke-free worksite than were white-collar workers throughout this 11-year study period. However, the overall coverage of workplace smoke-free policies increased from 46% to 71% nationwide, and the increasing trend was observed in all work groups (Plescia et al. 2005). Our study, conducted more recently (2003–2005), found that most of unionized trucking company workers are currently working in worksites with some kind of onsite smoking policy.

In our study, only 23% of nonsmokers and 10% of smokers reported that the policies were always enforced. These numbers suggest a relatively weak policy enforcement in this work setting. In the Plescia et al. (2005) study only 3% of workers reported that someone had violated the company policy in 2001–2002; however, when stratified by work groups, service and blue-collar workers reported slightly higher prevalence of noncompliance than did white-collar workers. In a CPS study conducted between 1999 and 2002, only 7% of nonsmokers reported that they experienced workplace SHS exposure (Pickett et al. 2006). It is interesting to note, however, that most of the trucking terminals in our study were located in the states or counties where a smoking ban was not enacted by law at the time we conducted the study. However, recently the number of states with smoking ban regulations increased remarkably. During 2003–2005, only about 15% of the workers in this study worked at the terminals located in the states or counties with smoking ban regulation (Table 1); but in 2008, about 66% of these workers (67% for nonsmokers and 64% for smokers) would have been covered by a state or county workplace smoking ban if they still worked at the same terminals.

Studies of serum cotinine are also consistent with the observation that blue-collar workers experience higher exposures to SHS than do white-collar workers. In the Third National Health and Nutrition Examination Survey (NHANES III) conducted from 1988 through 2002, blue-collar workers had higher serum cotinine levels than did other adults (Arheart et al. 2008; Pirkle et al. 2006; Wortley et al. 2002). The most recent study of NHANES III data suggested that although the serum cotinine levels for nonsmokers declined by 76% between 1988 and 2002 in all worker groups, blue-collar and service workers consistently had the highest levels of SHS exposure (Arheart et al. 2008), mainly because they work in areas with a higher prevalence of smokers.

Although numerous studies have focused on measuring levels of SHS exposure in the workplace in the service sector (e.g., bars and restaurants) (Hyland et al. 2000; Kiser and Boschert 2001; Repace et al. 2006; Weber et al. 2003), we identified only a few studies conducted in the 1980s that focused on quantifying levels in the transportation industry. In a study of workers in three railroads in 1982–1983, the median level of estimated vapor-phase nicotine (converted from particle-phase nicotine) among nonsmoking nonoffice workers was 0.10 μg/m^3^ (Hammond 1999; Schenker et al. 1990), which was much lower than the median level observed in our study (median of 0.93 μg/m^3^ for 183 nonsmoking nonoffice trucking workers). In 1983–1984, one of the railroads was revisited and vapor-phase nicotine levels were measured. The median nicotine level of nonsmoking engineers was 0.40 μg/m^3^ (Hammond 1999). This level was lower than the median level of 0.87 μg/m^3^ for nonsmoking truck drivers in our study, who worked at the similar condition as railroad engineers (small spaces, fewer other people). However, the nonsmoking office workers in the railroad were exposed to a greater nicotine level (median, 5.70 μg/m^3^) in 1983–1984, compared with the 20 office workers in our trucking population (median, 0.59 μg/m^3^).

In contrast to studies that used biomarkers, such as serum, urine, and salivary cotinine, to validate self-reported exposure to SHS (Emmons et al. 1994; George et al. 2006; Jenkins and Counts 1999; Kemmeren et al. 1994; Nondahl et al. 2005; Seccareccia et al. 2003), we measured vapor-phase nicotine concentrations in the breathing zone because of its ease of collection and because it avoided workplace biological sample collection and storage. In addition, the purpose of this study was to assess the SHS exposure pattern in the trucking industry, so we wanted only a measure of workplace exposure. Because biomarkers integrate exposures from all sources, they would not have been appropriate for this study. Moreover, the correlations between nicotine and commonly used biomarkers have been shown to be relatively good (LaKind et al. 1999; Leaderer and Hammond 1991).

In addition to evaluating the impact of smoking policy on workplace SHS exposure, we also attempted to assess the relationship between self-reported SHS exposure and vapor-phase nicotine levels among the trucking industry workers. Eisner et al. (2001) conducted a study to validate a SHS exposure survey, using the same personal badge sampling device as used in our study, among 50 nonsmoking asthmatic adults. They found a moderate correlation (*r* = 0.47) between self-reported SHS exposure duration and air nicotine concentration (median, 0.03 μg/m^3^) in the previous 7 days. A study conducted by O’Connor et al. (1995) among 415 nonsmoking pregnant women also demonstrated a similar correlation (*r* = 0.41) between SHS exposure duration and nicotine concentration (median, 0.1 μg/m^3^). These studies did not find a large impact of exposure intensity on the correlation between exposure duration and self-reported SHS exposure. In another study, Coghlin et al. (1989) found a strong correlation between an SHS exposure score (duration in hours × number of smokers × proximity of smokers) with log-transformed nicotine (*r* = 0.91) in 19 nonsmoking volunteers (nicotine level median, 2.0 μg/m^3^). In our study (median, 0.87 μg/m^3^ for nonsmokers), we found that the exposure duration combined with number of smokers were significant predictors of nicotine level after adjustment of confounders. It is possible that the intensity of exposure, as measured by number of smokers, might become more important when the nicotine level is higher.

In our multiple regression models, we found that work shift may also influence SHS exposure. Finally, we also found a statistically significant relationship between educational level and nicotine concentration in the multiple regression model for nonsmokers. Previous studies have suggested that higher prevalence of active smoking is related to lower educational level in the United States (Kanjilal et al. 2006) and many other countries (Gupta and Ray 2007), but limited studies focused on the association between educational level and SHS exposure. Kanjilal et al. (2006) reported that the smoking prevalence of the adults with lower educational level was significantly greater than the prevalence of those with higher educational level in the U.S. general population across 1971–2002. In our study, we did not find a significant association between active smoking and educational level among the smokers, possibly because of the relatively consistent social status in this blue-collar work group. However, we did find an association between lower educational level and workplace SHS exposure measured by personal sampling among the nonsmokers (Table 3). This implies that even within a blue-collar and relatively homogeneous population, educational status may still influence a nonsmoker’s exposure to SHS.

In summary, despite state and local movements toward smoke-free laws, this group of blue-collar workers was still exposed to workplace SHS as recently as 2005. Our findings suggest that most workplaces in this segment of the U.S. trucking industry have an official smoking policy. However, the workers’ perceived, rather than the official or state, policy was associated with measured SHS exposure levels among the nonsmokers. The self-reported duration and intensity of SHS exposure are relatively reliable among the nonsmokers in this work setting. In addition, factors such as job duties, work shift, and educational level might also be important predictors of workplace SHS exposure.

## Figures and Tables

**Table 1:** Baseline demographic, work-related, and health-related characteristics of participants by smoking status.

Variable	Nonsmokers (*n* = 203)	Smokers (*n* = 61)
Age (mean ± SD)	45.6 ± 8.9	45.9 ± 10.0
Years at school (mean ± SD)	12.9 ± 1.5	12.7 ± 1.1
Male [no. (%)]	189 (93.1)	60 (98.4)
White [no. (%)]	127 (62.6)	39 (63.9)
Driver [no. (%)]^a^	93 (45.8)	20 (32.8)
Work shift [no. (%)]		
Day	66 (32.5)	17 (27.9)
Evening	29 (14.3)	10 (16.4)
Night	27 (13.3)	10 (16.4)
Other/rotating	17 (8.4)	3 (4.9)
Official smoking policy [no. (%)]		
No policy/no restrictions	17 (8.4)	7 (11.5)
Certain indoor area	53 (26.1)	16 (26.2)
Outdoor only	133 (65.5)	38 (62.3)
Perception of workplace smoking policy [no. (%)]		
No policy/no restrictions	3 (1.5)	2 (3.3)
Certain indoor area	92 (45.3)	29 (47.5)
Outdoor only	90 (44.3)	25 (41.0)
State or county workplace smoking ban at sampling year [no. (%)]^b^		
No	173 (85.2)	51 (83.6)
Yes	30 (14.8)	10 (16.4)
Bothered by tobacco smoke at work in the past 7 days [no. (%)]	78 (38.4)	1 (1.6)
Experience irritation after exposure to tobacco smoke [no. (%)]		
Any irritation	49 (24.1)	6 (9.8)
Eye irritation	35 (17.2)	5 (8.2)
Nose irritation	32 (15.8)	4 (6.6)
Throat irritation	31 (15.3)	5 (8.2)

a Including long-haul drivers and P&D drivers.

b State/county where the workers’ trucking terminals located; sampling years: 2003–2005.

**Table 2:** Nicotine concentration (μg/m^3^) by demographic, work-related, and health-related variables among U.S. trucking industry workers, 2003–2005.

	Nonsmokers				Smokers			
Characteristic	*n*	Median (IQR)	Range	Adjusted *p*-value^a^	*n*	Median (IQR)	Range	Adjusted *p*-value^a^
Overall	203	0.87 (0.38–2.26)	0–56.9		61	5.96 (2.49–14.31)	0–101.7	

Race								
White	127	1.07 (0.39–2.72)	0–37.0	0.78	39	5.70 (2.85–13.8)	0–101.7	0.37
Nonwhite	30	0.87 (0.42–2.24)	0.07–56.9		9	1.85 (1.00–8.02)	0.3–51.2	

Education (years)								

> 12	72	0.76 (0.34–2.33)	0–56.9	0.33	20	3.77 (1.22–15.7)	0–101.7	0.97
≤ 12	115	0.90 (0.42–2.17)	0–37.0		36	5.83 (3.38–12.8)	0–86.5	

Job title^b^								

Nondriver	110	0.86 (0.43–2.29)	0–37.0	0.07	41	7.56 (1.85–14.5)	0–101.7	0.34
Driver	93	0.87 (0.34–2.12)	0–56.9		20	4.61 (3.38–12.3)	1.44–51.2	

Work shift								

Day	66	1.03 (0.39–2.79)	0–37.0	0.06	17	4.56 (1.99–10.8)	0–21.6	0.22
Evening	29	1.00 (0.38–1.80)	0.15–56.9		10	9.93 (0.68–39.9)	0.3–101.7	
Night	27	1.04 (0.48–3.12)	0–10.9		10	5.13 (3.36–7.21)	0–16.9	
Rotating/other	17	0.73 (0.32–2.24)	0.15–6.57		3	2.47 (1.00–68.4)	1–68.4	

Official smoking policy								

No policy/no restrictions	17	1.00 (0.62–2.58)	0.31–6.59	0.81	7	8.83 (1.00–45.0)	0.55–51.2	0.88
Certain indoor area	53	0.80 (0.43–1.48)	0–56.9		16	8.22 (2.70–18.5)	0–86.5	
Outdoor only	133	0.90 (0.35–2.38)	0–37.0		38	5.13 (2.85–13.8)	0.3–101.7	

Perception of workplace smoking policy								

No policy/no restrictions	3	3.50 (2.56–5.64)	2.56–5.64	0.04^c^	2	20.3 (20.1–20.4)	20.1–20.4	0.11^c^
Certain indoor area^d^	92	0.79 (0.36–1.75)	0.01–56.9		29	4.46 (1.98–8.69)	0–86.5	
< 4 pallets	28	0.72 (0.40–1.97)	0.06–56.9		4	4.68 (2.69–6.86)	1.98–7.75	
4–10 pallets	27	0.77 (0.35–1.80)	0.25–12.1		11	4.46 (0.68–16.9)	0–68.4	
> 10 pallets	37	0.79 (0.32–1.48)	0.01–37.0		14	4.39 (2.49–8.83)	0–86.5	
Outdoor only	90	1.09 (0.43–2.79)	0–15.3		25	9.34 (2.47–14.3)	0.36–51.2	

State or county workplace smoking ban								

No workplace ban	173	0.90 (0.39–2.28)	0–56.9	0.36	51	7.75 (2.47–14.5)	0–101.7	0.75
Workplace ban	30	0.71 (0.28–1.97)	0.11–9.46		10	4.34 (2.49–5.70)	0.3–86.5	

Time spent/average no. people smoking in the restricted area in the previous 2 days								

≤ 30 min/≤ 3 people	147	0.81 (0.37–2.56)	0–56.9	0.09	36	4.52 (2.23–12.3)	0–101.7	0.60
≤ 30 min/> 3 people	25	0.80 (0.34–1.48)	0.02–12.1		8	8.68 (2.69–15.2)	0.55–21.6	
> 30 min/≤ 3 people	11	1.00 (0.32–2.38)	0.11–6.91		5	7.75 (1.78–16.9)	1.44–45.0	
> 30 min/> 3 people	19	1.13 (0.47–3.42)	0.01–37.0		12	9.78 (4.46–17.2)	0.62–43.3	

Cigarettes smoked in the previous 2 days								

≤ 20	—	—	—	—	29	2.95 (1.00–7.21)	0–31.3	0.002
> 20	—	—	—		31	11.7 (4.56–21.6)	1–101.7	

Bothered by tobacco smoke at work in the previous 7 days								

Never	51	0.74 (0.37–2.56)	0–15.3	0.79	34	4.52 (2.47–9.34)	0–86.5	0.35
Ever	78	1.07 (0.47–2.38)	0–37.0		1	21.6 (NA)	—	

Eye irritation								

No	109	0.87 (0.38–2.14)	0.01–15.3	0.08	40	7.48 (2.23–14.0)	0–101.7	0.30
Yes	35	1.22 (0.50–3.30)	0–12.1		5	5.60 (3.56–7.56)	1.85–43.3	

Nose irritation								

No	114	0.89 (0.38–2.14)	0.01–15.3	0.21	39	7.75 (2.47–14.3)	0–101.7	0.52
Yes	32	0.95 (0.49–3.54)	0–37.0		4	4.58 (2.77–24.4)	1.98–43.3	

Throat irritation								

No	113	0.90 (0.38–2.05)	0.01–15.3	0.26	40	7.48 (2.23–14.1)	0–101.7	0.30
Yes	31	0.81 (0.50–3.00)	0–12.1		5	5.60 (3.56–7.56)	1.85–43.3	

Abbreviations: IQR, interquartile range; NA, not applicable.

a Wilcoxon rank-sum test (dichotomous variables) or Kruskal–Wallis test (variables with more than two categories), adjusted for trucking terminal.

b Job duty that the workers actually did during the sampling days.

c *p*-Value of Kruskal–Wallis test comparing “no policy,” “certain indoor area,” and “outdoor only.”

d One pallet = 4 ft × 4 ft = 16 ft^2^.

**Table 3:** Regression coefficients of questionnaire-based measures associated with nicotine concentration (μg/m^3^) for nonsmoking trucking industry workers.^a^

	Univariate (crude)^b^		Age- and terminal-adjusted^c^		Multivariable-adjusted^d^	
Variable	Slope (SE)	*p*-Value	Slope (SE)	*p*-Value	Slope (SE)	*p*-Value
Time spent/number of people smoking in smoking area,^e^ > 30 min/> 3 people vs. others	0.26 (0.59)	0.67	1.05 (0.55)	0.06	1.31 (0.55)	0.02
Perception of workplace smoking policy						
Smoking allowed at certain indoor area	Reference	—	Reference	—	Reference	—
Smoking allowed outdoors only	0.57 (0.34)	0.10	0.21 (0.28)	0.45	0.35 (0.27)	0.19
No policy/no restrictions	2.43 (0.78)	0.002	2.59 (0.99)	0.01	2.89 (0.93)	0.002
Job title, nondrivers vs. drivers^f^	0.34 (0.32)	0.28	0.58 (0.26)	0.02	0.70 (0.27)	0.01
Work shift, night shift vs. others	0.37 (0.54)	0.50	0.79 (0.44)	0.07	0.71 (0.38)	0.06
Education, years at school	−0.11 (0.09)	0.23	−0.12 (0.09)	0.16	−0.18 (0.09)	0.04

a Linear regression analyses using robust variances; *n* = 198 after removing influential outliers.

b Univariate analyses (crude results).

c Adjusted for trucking terminal and age (quartile).

d Adjusted for trucking terminal, age (quartile), and all the variables listed in the table; model intercept (SE) = 1.88 (1.35), *R*^2^ = 0.52.

e Time (minutes) spent in smoking allowed areas in the past two days (personal badge sampling days)/average numbers of people smoking in the smoking allowed areas that the subject entered in the past two days (personal badge sampling days).

f Job duty that the workers actually did during the sampling days.
